# Hereditary multiple exostoses caused by a chromosomal inversion removing part of *EXT1* gene

**DOI:** 10.1186/s13039-023-00638-0

**Published:** 2023-05-22

**Authors:** Angelos Alexandrou, Nicole Salameh, Ioannis Papaevripidou, Nayia Nicolaou, Panayiotis Myrianthopoulos, Andria Ketoni, Ludmila Kousoulidou, Anna-Maria Anastasiou, Paola Evangelidou, George A. Tanteles, Carolina Sismani

**Affiliations:** 1 Cytogenetics and Genomics Department, The Cyprus Institute of Neurology and Genetics, 6 Iroon Avenue, 2371, Ayios Dometios, PO Box 23462, 1683 Nicosia, Cyprus; 2 Clinical Genetics Department, Cyprus Institute of Neurology and Genetics, Nicosia, Cyprus

**Keywords:** Hereditary multiple exostoses, *EXT1*, Complex chromosomal rearrangement

## Abstract

**Background:**

Hereditary multiple exostoses (HME) is an autosomal dominant skeletal disorder characterized by the development of multiple, circumscript and usually symmetric bony protuberances called osteochondromas. Most HME are caused by *EXT1* and *EXT2* loss of function mutations. Most pathogenic mutations are nonsense followed by missense mutations and deletions.

**Case presentation:**

Here we report on a patient with a rare and complex genotype resulting in a typical HME phenotype. Initial point mutation screening in *EXT1* and *EXT2* genes by Sanger sequencing did not reveal any pathogenic variants. The patient along with the healthy parents was subsequently referred for karyotype and array-Comparative Genomic Hybridization (CGH) analyses.

Chromosomal analysis revealed two independent de novo apparently balanced rearrangements: a balanced translocation between the long arms of chromosomes 2 and 3 at breakpoints 2q22 and 3q13.2 and a pericentric inversion with breakpoints at 8p23.1q24.1. Both breakpoints were confirmed by Fluorescence In Situ Hybridization (FISH). Subsequently, array-CGH revealed a novel heterozygous deletion within the *EXT1* gene at one of the inversion breakpoints, rendering the inversion unbalanced. The mode of inheritance, as well as the size of the deletion were further investigated by Quantitative Real-time PCR (qPCR), defining the deletion as de novo and of 3.1 kb in size, removing exon 10 of *EXT1*. The inversion in combination with the 8p23.1 deletion most likely abolishes the transcription of *EXT1* downstream of exon 10 hence resulting in a truncated protein.

**Conclusions:**

The identification of a rare and novel genetic cause of HME, highlights the importance of additional comprehensive investigation of patients with typical clinical manifestations, even when *EXT1* and *EXT2* mutation analysis is negative.

## Background

Hereditary multiple exostoses (HME) is an autosomal dominant skeletal disorder characterized by the development of multiple, circumscript, occasionally painful and usually symmetric bone protuberances called osteochondromas [1]. Amongst other problems, HME can lead to a reduction in skeletal growth, secondary bone deformities, restricted joint motion, short stature and compression of peripheral nerves [2].

HME is a genetically heterogeneous condition with an estimated penetrance of approximately 96% in females and 100% in males and displays great inter- and intra-familial variability in phenotypic expression. The severity of the phenotype is defined by the number of exostoses, the extent of deformities, the intensity of pain and functional limitations [3]. HME is known to be caused by *EXT1* (exostosin-1)*,* and *EXT2* loss of function mutations [4], the majority of which are nonsense and frameshift mutations [5]. Missense, splice-site mutations and deletions also constitute a large proportion of HME genetic determinants [5, 6]. It has been estimated that approximately 90% of the HME cases are caused by mutations in one of the EXT genes with different studies reporting a range of 4% to 33% of cases without any causative EXT variant (reviewed in [2, 7, 8]). This led to the speculation that the HME phenotype may also be caused by other genes, somatic mosaicism and mutations within intronic regions [2].

The prevalence of *EXT1* and *EXT2* mutations appears to differ among HME patients of different ethnicities, with the *EXT1* prevailing in European populations, accounting for 65% of HME [9] and *EXT2* being more frequent in Chinese and Saudi-Arabian patients [2, 10].

Genes that belong to the EXT family encode glycosyltransferases, which in turn participate in the synthesis of heparin sulfate proteoglycans (HSPGs) [11, 12]. HSPGs regulate the formation of bone and cartilage by interacting with bone proteins [8]. Recent studies have shown that EXT1 and EXT2 proteins exist in a hetero-dimeric form, with the main catalytic activity attributed to the EXT1 glycosyltransferase domain, which is consistent with the higher prevalence of *EXT1* mutations in HME patients [13].

Here, we report on a rare complex unbalanced chromosomal rearrangement leading to a typical and severe HME phenotype. Our data demonstrate the impact of apparently balanced and unbalanced chromosomal rearrangements on the function of implicated genetic loci and highlights the importance of cytogenetic investigation for genetic diagnosis of patients with known and well-characterized genetic disorders for which mutation analysis of known causative genes is negative.

## Case presentation

The patient is a 12-year-old girl born to healthy non-consanguineous parents. She received a preliminary diagnosis of HME, based on clinical examination and on the presence of multiple osteochondromas.

The patient’s father has a history of hypertrophic cardiomyopathy. There is also a family history of Friedreich ataxia on the paternal side.

This was the mother’s second pregnancy following a natural conception, complicated by a maternal bleed at three months of gestation. No intercurrent infection or known exposure to teratogens were reported. Antenatal scans were normal. The proband was born at 38 weeks of gestation, perinatal period was unremarkable, with a birth weight of 2.5 kg and normal APGAR score. She had mild reflux in infancy, walked at the age of 14 months and had a slight speech delay. At the age of four, she developed a lump just above her left knee. Subsequent X-rays revealed an exostosis and further exostoses emerged over the following 2–3 years.

At the time of her initial evaluation in clinical genetics clinic (at the age of 11 years), she had multiple exostoses and was under the care of orthopaedic surgeons. She had a history of leg length discrepancy. On examination, her occipital frontal circumference (OFC) was 54.5 cm (64th centile), her height was 145 cm (55th centile) and her weight was 33 kg (32nd centile). She had multiple exostoses at her fingers, right humerus, elbows and knees. She had mild tenderness over her middle spine at the level of the lower scapular edges. Her abdomen was soft and non-tender with no evidence of renal angle tenderness. She had a valgus deformity of the left knee particularly with a mild degree of leg-length discrepancy. Cardiovascular and neurological evaluations were unremarkable. Assessment by an educational psychologist revealed no concerns and no learning difficulties were observed.

### Genetic study

Mutations in *EXT1* and *EXT2* genes were previously excluded by Sanger sequencing.

#### Conventional cytogenetic findings

**Fig. 1:**
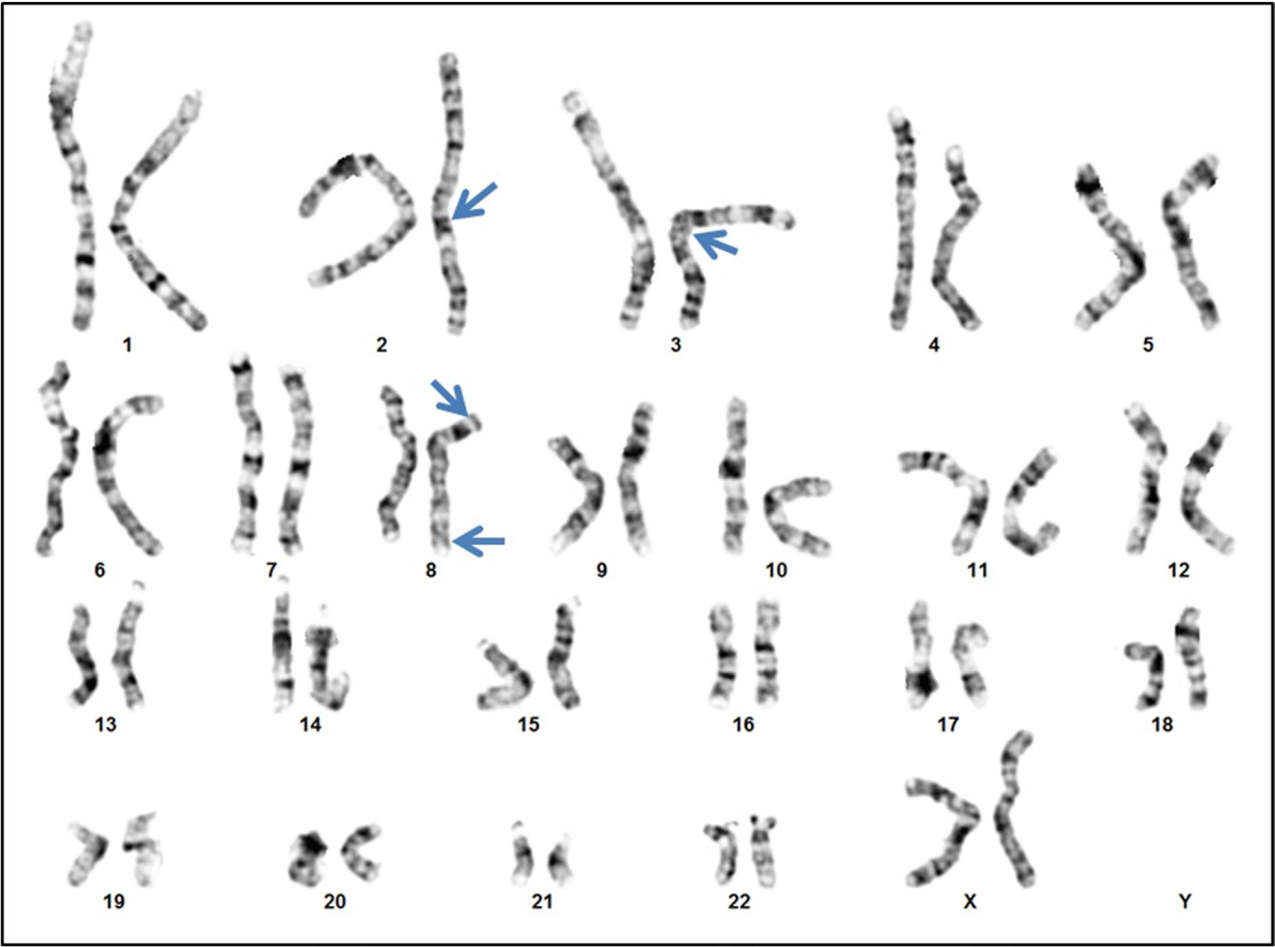
Karyotype showing an apparently balanced translocation between the long arms of chromosomes 2 and 3 and the pericentric inversion on chromosome 8 as indicated by the arrows [46,XX,t(2;3)(q22;q13.2),inv(8)(p23.1q24.1)dn]

Conventional cytogenetic G Banding analysis was carried-out on peripheral blood lymphocytes using standard cytogenetic methodologies with an average resolution of 550 bands. Chromosomal analysis revealed a female karyotype with two rearrangements in the proband: an apparently balanced translocation between the long arms of chromosomes 2 and 3 at breakpoints 2q22 and 3q13.2 respectively and a pericentric inversion on chromosome 8 with breakpoints 8p23.1 and 8q24.1, with the initial karyotype designated as 46,XX,t(2;3)(q22;q13.2),inv(8)(p23.1q24.1) (Fig. 1). Chromosomal analyses of the parents did not reveal any aberrations.

#### Molecular findings

DNA samples of the patient and both biological parents were obtained by isolation from 2 ml peripheral blood using the Qiagen Midi Kit according to the manufacturer’s protocol (Qiagen, Valencia, CA).

**Fig. 2:**
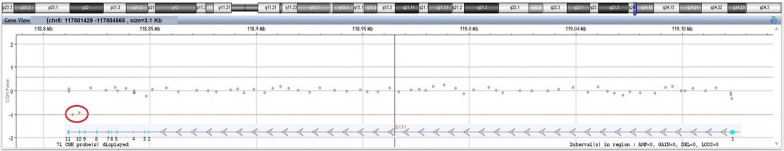
Array-CGH profile indicating a copy number loss at 8q24.11 as illustrated by the blue box and shown by the two oligomers circled in red. The loss is located within exon 10–11 (light blue lines) of the *EXT1* gene

Array-based Comparative Genomic Hybridization (array CGH) was performed using SurePrint ISCA array (Agilent Technologies Inc., Santa Clara) with 60,000 oligos according to the manufacturer’s recommendations. The array was scanned at 2 mm resolution using the Agilent DNA microarray scanner and fluorescent ratios were calculated using the Agilent Cytogenomics-version 5.1 (Agilent Technologies Inc., Santa Clara). A copy number loss of approximately 3.1 kb was detected on chromosomal band 8q24.11 at position 117,801,429 (start) to 117,804,565 (end) (GRCh38) (Fig. 2). No imbalance was detected at either chromosome 2 or 3 breakpoints.

**Fig. 3:**
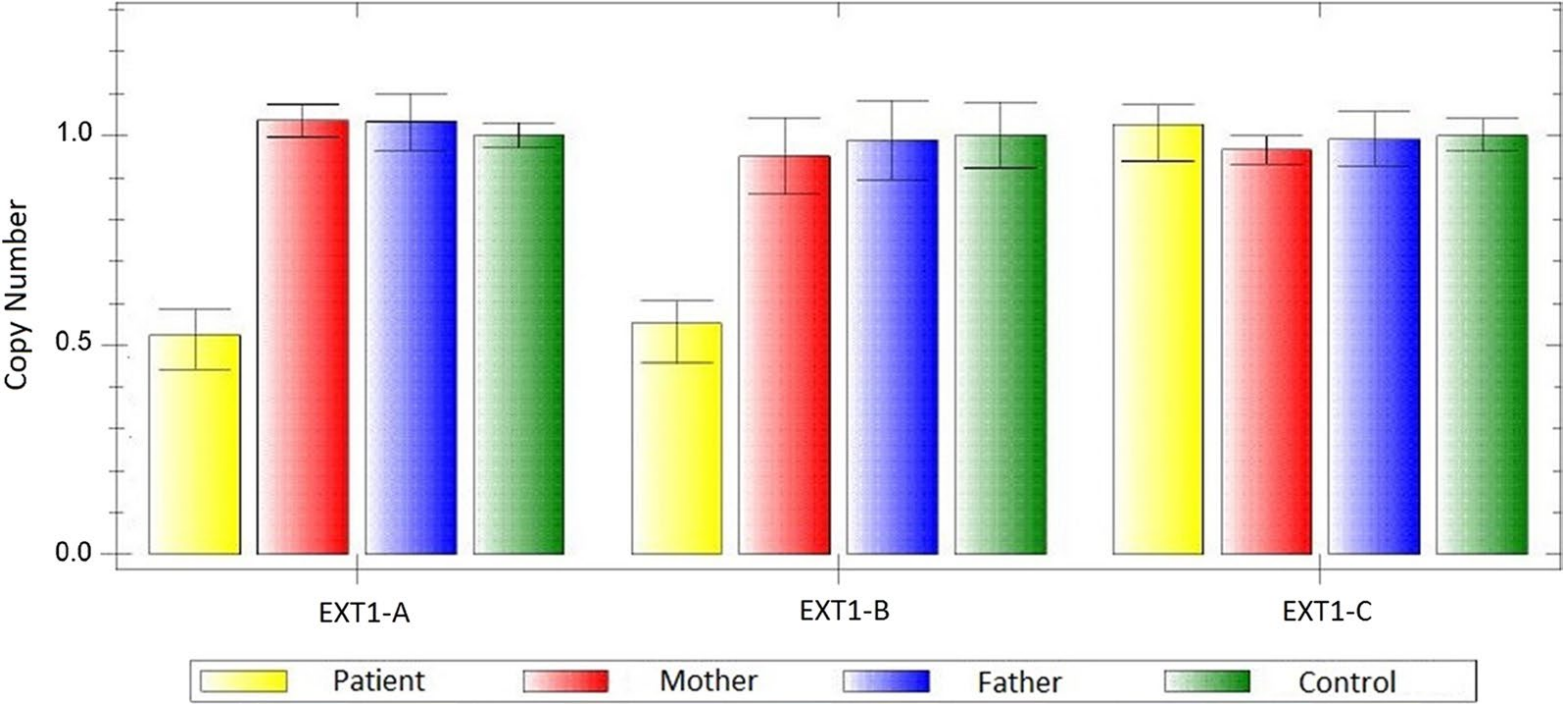
qPCR bar graph illustrates the confirmation and extent of the *EXT1* deletion. Primer EXT1-A (located between exons 10 -11) and EXT1-B (exon 10) showing a deletion as indicated by the copy number values of 0.5 and EXT1-C (exon 9) normal with a value of 1 compared to the normal female control. The parents show normal copy number for the same regions

**Fig. 4:**
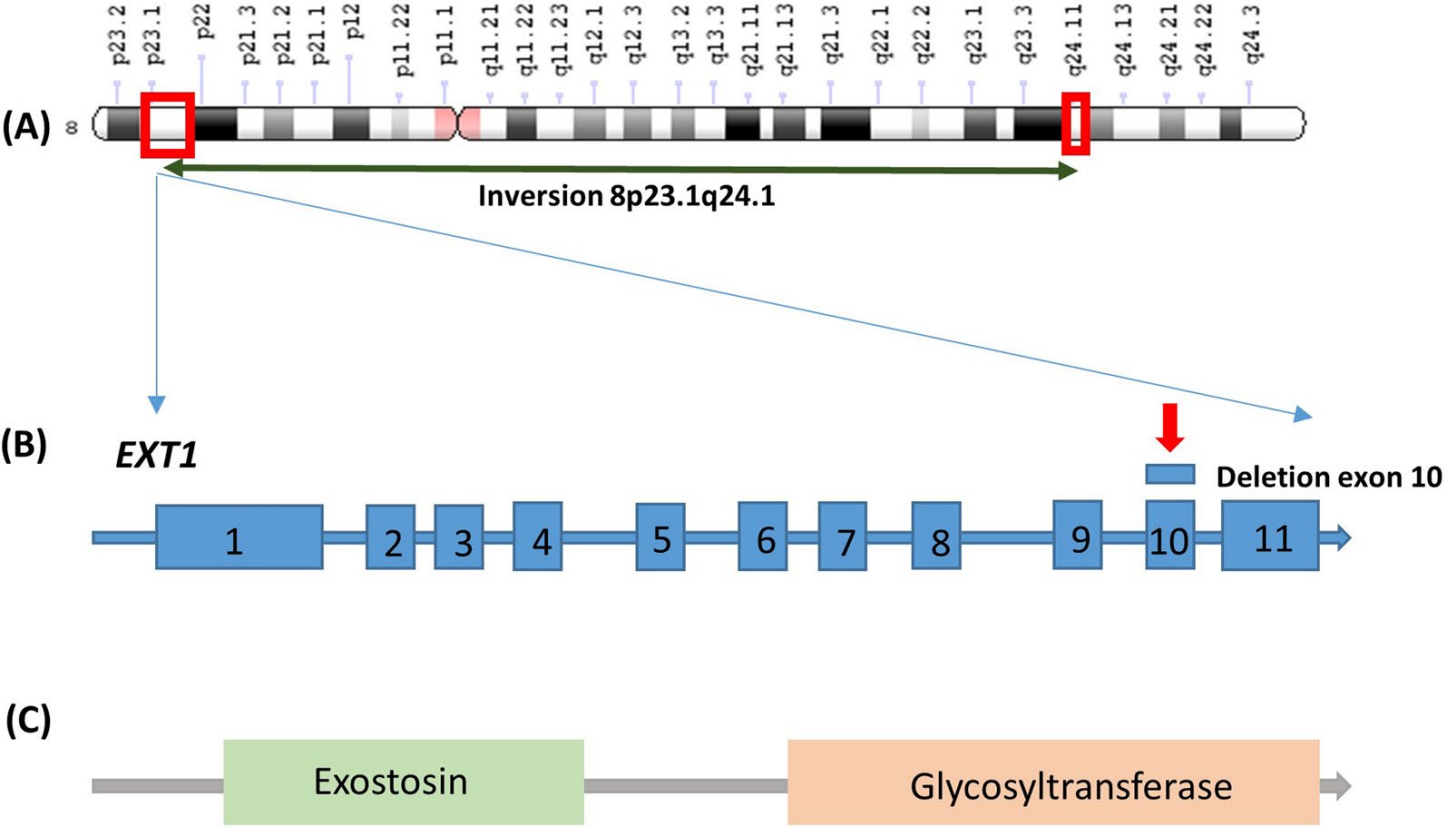
**A** Chromosome 6 ideogram illustrating the inversion breakpoints (shown in red boxes) **B** *EXT1* gene structure: deletion in exon 10 shown in red arrow. **C** Functional domains of the EXT1 protein encoded by the normal *EXT1* gene

Quantitative Real-Time Polymerase Chain Reaction analysis (qPCR) was carried-out using specifically designed primer pairs (Metabion, Germany), within the deleted region (primer sequences are available upon request). qPCR was performed as described elsewhere[14]. PCR, detection and fluorescent data analysis were carried out on the CFX96 real-time C1000 thermal cycler (Biorad, Hercules, USA) using the Sso Fast Evagreen Supermix (Biorad). qPCR confirmed the deletion and redefined the breakpoints to position 117,801,429–117,805,595 (Fig. 3). The deleted region lies within exostosin glycosyltransferase gene (*EXT1*, OMIM # 608,177) [15] removing exon 10 (Fig. 4).qPCR of the parents did not reveal any copy number aberrations; therefore, the patient’s deletion was de novo.

#### Molecular cytogenetic findings

**Fig. 5:**
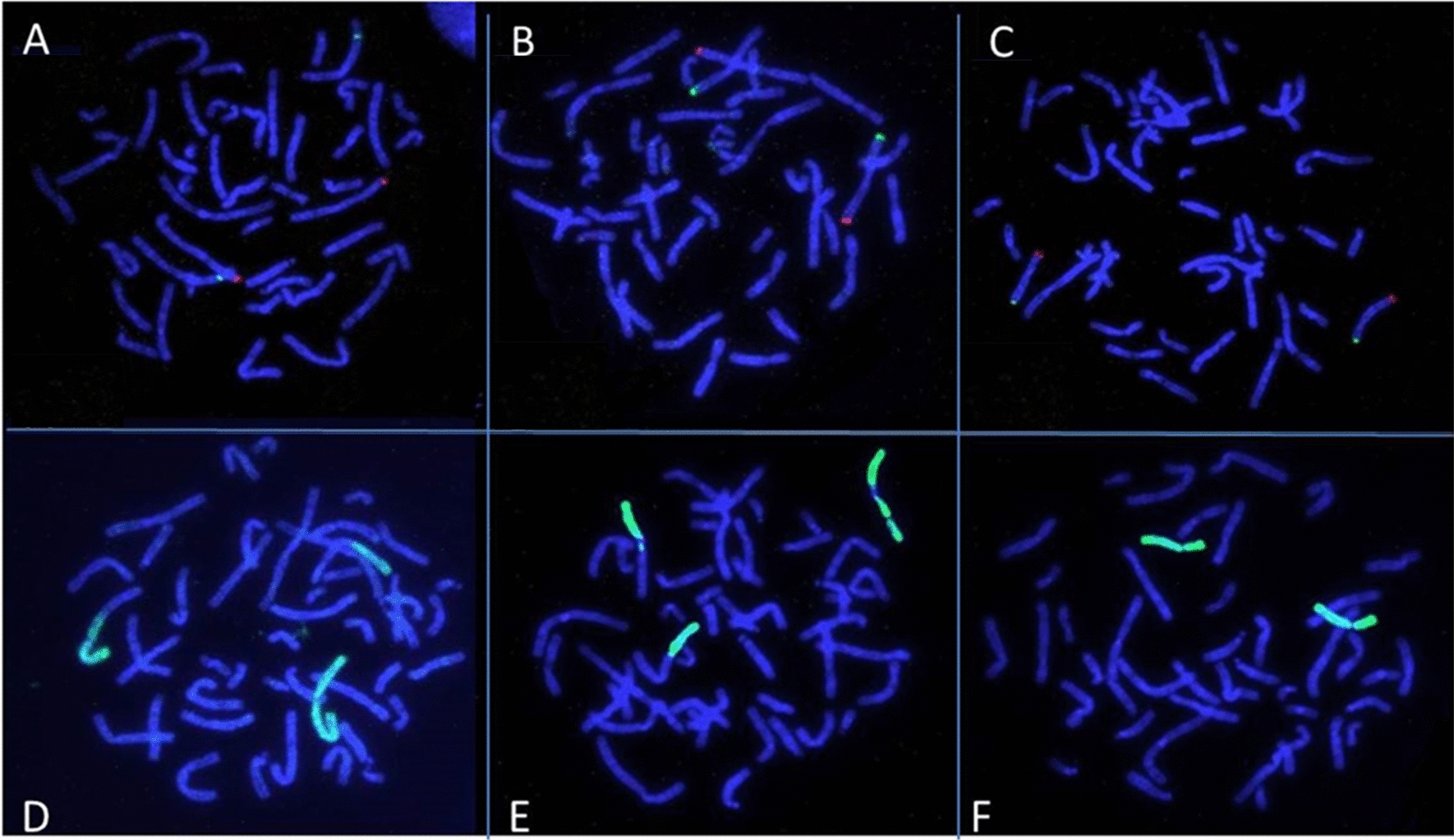
FISH images using locus specific probes of chromosome 2 (**A**), subtelomere-specific probes of chromosomes 3 (**B**) and 8 (**C**) and Whole Chromosome Paints for chromosomes 2 (**D**), 3 (**E**) and 8 (**F**). FISH analysis confirmed the translocation between chromosomes 2 and 3 and the inversion of chromosome 8, excluding the involvement of other chromosomes in the rearrangements

The translocation, as well as, the pericentric inversion were further investigated with Fluorescence In Situ Hybridization (FISH) using subtelomere-specific probes for chromosomes 2, 3 and 8 (Cytocell. Co), according to the manufacturer’s recommendations. FISH analysis confirmed the detected chromosomal rearrangements and excluded the involvement of other chromosomes (Fig. 5).

## Discussion and conclusions

Mutations in either the *EXT1* or *EXT2* gene on chromosome 8q24.11 have been shown to cause HME accounting for approximately 70–95% of cases [16, 17]. Intragenic deletions, involving single or multiple *EXT1* (or *EXT2*) exons are found in approximately 10% of tested patients [18]. It has been observed that in general, patients with *EXT1* mutations exhibit more severe manifestations than *EXT2* mutation carriers or patients without any causative mutations detected during genetic testing [9, 19].

In this study, we have performed conventional cytogenetic analysis of a patient with typical HME [20] along with her parents, and identified an apparently balanced de novo translocation between chromosomes 2 and 3 and an apparently balanced inversion on chromosome 8. Further investigation revealed an intragenic *EXT1* deletion, possibly resulting from the pericentric inversion on chromosome 8 and located within its breakpoint region. Association of structural rearrangements with HME has been previously reported in the literature. One of the earliest reports describe a female carrier of a balanced translocation t(8;11)(q24.11;p15.5) manifesting a classical HME phenotype with no other clinical manifestations [21]. In another report, two patients manifesting HME among other phenotypic abnormalities, were found to carry balanced and unbalanced translocations affecting the 8q24 region [22]. In a large family, eight members of three generations carried a reciprocal translocation t(8;19)(q24.11;q13.13), disrupting the first intron of *EXT1* gene and exhibited a moderately severe HME along with male infertility, recurrent miscarriages and slightly reduced stature [23].

The location of the breakpoints in apparently balanced structural chromosomal rearrangements including inversions, is an important determinant of any potential clinical consequences. When coding genes are affected, either by direct disruption or by position effect, abnormal phenotypes may occur [24, 25]. There are several examples in the literature illustrating this effect, such as the study by Watson et al., where a patient with hand-foot-genital syndrome was found to carry a 7p15 inversion, displacing the enhancer sequences from the HoxA cluster, which is responsible for body patterning amid embryo development [26]. Another interesting mechanism was demonstrated by Lettice et al. [27], suggesting that gain of long-range cis-regulatory elements may be a frequent mechanism that causes phenotypic anomalies in carriers of apparently balanced inversions. In a study by Colovati et al. a patient manifesting Marfan syndrome is found to carry a deletion encompassing *FBN1* gene, within the context of a complex translocation involving three chromosomes, with three breakpoints on chromosome 15 where *FBN1* gene is located [28].

Inversions can also co-exist with or predispose to other rearrangements, causing complex phenotypes [29, 30]. In our case, the balanced t(2;3) in the patient most likely does not contribute to the manifestation of HME in the proband, as no copy number gains or losses were detected within chromosomes 2 or 3. The breakpoints of the translocation were not further investigated as no critical regions related to the patient’s phenotype were found to be located near the breakpoints. The inversion on chromosome 8 in combination with the 8p24.11 deletion most likely abolishes the transcription of *EXT1* downstream of exon 10 hence resulting in a truncated protein, affecting the glycosyltransferase domain of the polypeptide (Fig. 5). Even though we assume that the deletion is a result of the inversion, we cannot exclude the possibility that is has occurred independently on the non-inverted homologous chromosome. The FISH analysis performed in our patient has only confirmed the complex rearrangements detected by karyotype, but did not specifically target the deleted region. In any case, since *EXT1* mutations and deletions have a dominant effect, the clinical consequences of a loss within the *EXT1* region would be the same, regardless the mechanism by which they have occurred. Further investigation of the deletion breakpoints by FISH and sequencing approaches would shed light on possible effects on the direction and efficiency of transcription and would contribute to more accurate predictions of a possible impact of the rearrangement.

Our study has demonstrated the crucial role of karyotype analysis in elucidating the genetic basis of severe clinical phenotypes, even when a specific causative gene is suspected. Targeted mutation screening may not be sufficient as a first-tier test and a possible disruption or copy number alteration within candidate genes by large-scale chromosomal rearrangements should be considered as a potential disease mechanism.

A rare and novel genetic cause of HME is presented, highlighting the importance of additional comprehensive cytogenetic examination of the *EXT1* and *EXT2* genes, when the mutation analysis is negative. A combination of conventional cytogenetics, molecular cytogenetics and molecular genetics analyses could be very enlightening and valuable in similar cases.

## Data Availability

The datasets used and/or analyzed during the current study are available from the corresponding author on reasonable request.

## Abbreviations

HME: Hereditary multiple exostoses
Array-CGH: Array comparative genomic hybridization
FISH: Fluorescence in situ hybridization
qPCR: Quantitative real-time polymerase chain reaction
OFC: Occipital frontal circumference
HSPGs: Heparin sulphate proteoglycans
